# Gene therapy in polycystic kidney disease: A promising future

**DOI:** 10.1515/jtim-2024-0021

**Published:** 2025-01-10

**Authors:** Cheng Xue, Jiayi Lv, Bo Yang, Shuqin Mei, Jing Xu, Xinming Li, Liming Zhang, Zhiguo Mao

**Affiliations:** Division of Nephrology, Shanghai Changzheng Hospital, Second Military Medical University (Naval Medical University), Shanghai 200003, China; Internal Medicine III (Nephrology), Naval Medical Center of PLA, Naval Medical University, Shanghai 200433, China; Department of Nephrology, Zhabei Central Hospital of Jing’an District, Shanghai 200120, China

**Keywords:** polycystic kidney disease, gene therapy, antisense oligonucleotides, Crispr-Cas9, adeno-associated virus vector

## Abstract

Polycystic kidney disease (PKD) is a genetic disorder marked by numerous cysts in the kidneys, progressively impairing renal function. It is classified into autosomal dominant polycystic kidney disease (ADPKD) and autosomal recessive polycystic kidney disease (ARPKD), with ADPKD being more common. Current treatments mainly focus on symptom relief and slowing disease progression, without offering a cure. Recent advancements in gene editing technologies, such as CRISPR-Cas9, have introduced new therapeutic possibilities for PKD. These approaches include miR-17 antisense oligonucleotides, adenovirus-mediated gene knockdown, Pkd1 gene or polycystin -1 C-terminal tail enhancement therapy, and 3′-UTR miR-17 binding element by CRISPR-Cas9, which have shown potential in animal models and early clinical trials. Specifically for ARPKD, strategies like antisense oligonucleotide therapy targeting c-myc and CRISPR/ Cas9 knockdown of the P2rx7 gene have shown promise. Despite facing challenges such as technological limitations, ethical and legal issues, and high costs, gene therapy presents unprecedented hope for PKD treatment. Future interdisciplinary collaboration and international cooperation are essential for developing more effective treatment strategies for PKD patients.

## Introduction

Polycystic kidney disease (PKD) is a genetic condition primarily marked by the development of numerous cysts within the kidneys, leading to progressive renal impairment over time.^[1]^ This disease manifests in two key forms: autosomal dominant polycystic kidney disease (ADPKD) and autosomal recessive polycystic kidney disease (ARPKD), distinguished by their patterns of inheritance. ADPKD, the more prevalent variant, affects approximately 1 in 1000 to 2500 individuals, with symptoms typically emerging in adulthood.^[2]^ Conversely, ARPKD is less common, occurring in about 1 in 20,000 births, and often presents in infancy or childhood. Beyond its impact on kidney function, PKD may also adversely affect other organs, including the liver, pancreas, and cardiovascular system, underscoring the systemic nature of the disorder.^[3]^ Current treatment strategies primarily aim to mitigate symptoms and decelerate the disease’s progression, with Tolvaptan being the only medication approved for slowing kidney volume growth and renal function decline in ADPKD patients.^[4]^ However, its use is tempered by side effects such as polyuria and elevated liver enzymes, necessitating careful medical supervision.^[3]^ The advent of gene editing technologies and advancements in molecular biology in recent years has sparked new optimism for PKD treatment, particularly for ADPKD.^[5]^ The potential of gene therapy to directly alter or regulate the genes responsible for PKD offers a groundbreaking approach to tackling the disease’s underlying pathogenesis. This review will explore the latest developments in gene therapy research and treatment for both ADPKD and ARPKD, examining the promising avenues, inherent challenges, and the prospective future of these innovative treatments.

## PKD genes, pathogenesis, clinical manifestations, and prognosis

The genetic underpinnings of PKD, particularly ADPKD, outline a sophisticated pathophysiological landscape. At the heart of ADPKD are two primary genes, *Pkd1* and *Pkd2*. *Pkd1* resides on the short arm of chromosome 16 (16p13.3), where the presence of six pseudogenes with high G-C content complicates mutation detection efforts.^[6]^ Conversely, *Pkd2* is found on the long arm of chromosome 4 (4q22-23). Together, mutations in Pkd1 and Pkd2 account for 78% and 15% of ADPKD cases, respectively, leaving 7% of cases undetected by these two genes.^[6]^ *Pkd1*’s expansive gene length, featuring 46 exons, encodes polycystin-1 (PC1)—a protein integral to calcium signaling and essential for maintaining tubular epithelial cell polarity and function, with its 4303 amino acid residues. Meanwhile, *Pkd2* gene produces polycystin-2, which collaborates with polycystin-1 to regulate cellular calcium levels. Disruptions in these genes impair the functions of their protein products, promoting abnormal cell proliferation, differentiation, inflammation, epigenetics, and fluid secretion, culminating in cyst development and progressive renal decline.^[7,8]^ The widespread distribution of *Pkd1* mutations across its coding region further complicates the genetic landscape of ADPKD.

Mayo PKD gene database has documented the genetic diversity within the PKD community, reporting mutations in 22,077 families—322 in *Pkd1* and 278 in *Pkd2*. Our analysis^[9]^ of 98 Chinese ADPKD families revealed 93 pathogenic mutations, 85 in *Pkd1* and 8 in *Pkd2*, with 55 (59.1%) being newly reported mutations and 9 de novo mutations. Then, our subsequent search among 44 ADPKD families in China identified 37 *PKD2* gene variants, including 18 nonsense mutations, 10 frameshift mutations, 4 missense mutations, and 5 splice-site mutations, 11 of which were reported for the first time.^[10]^

The advent of high-throughput gene sequencing has unveiled additional genes implicated in ADPKD and ARPKD, such as *GANAB* and *DNAJB11*, enhancing our comprehension of PKD’s genetic diversity. *GANAB* mutations are associated with atypical ADPKD presentations, while *DNAJB11* has unveiled the significance of endoplasmic reticulum responses in PKD’s progression. These discoveries not only deepen our understanding of PKD’s pathogenesis but also spotlight crucial targets for developing focused gene therapy approaches, offering new avenues for personalized and precision medicine in combating PKD.

Symptoms of ADPKD usually appear in adulthood, including hypertension, hematuria, flank pain, and progressive renal impairment. Patients may also develop cysts in other organs such as the liver, pancreas, and spleen, and face an increased risk of cardiovascular abnormalities, including aneurysms.^[2]^ PKD1 mutations are typically associated with a more severe form of the disease, with symptoms such as hypertension, hematuria, flank pain, and kidney enlargement generally presenting earlier in life. Patients with *PKD1* mutations tend to progress to endstage renal disease (ESRD) faster.^[11]^ In contrast, PKD2 mutations usually result in a milder form of the disease with a later onset of symptoms. Renal function declines more slowly in these patients. Extra-renal manifestations, such as liver cysts, are less common compared to *PKD1* mutations.^[11,12]^

The severity and rate of progression in ADPKD can vary significantly, influenced by the specific mutation type and location, as well as other genetic and environmental factors. Patients with *PKD1* mutations especially truncation mutations generally have a poorer prognosis due to the earlier onset and rapid progression of the disease, with most developing ESRD by their 50s, necessitating dialysis or kidney transplantation.^[11]^ On the other hand, patients with *PKD2* mutations tend to have a better prognosis, with slower disease progression. ESRD typically occurs later, often in the 60s or beyond, allowing for a longer period of preserved kidney function.^[11]^

ARPKD primarily stems from mutations in the *PKHD1* gene, located at 6p12.2, which codes for the protein fibrocystin. Despite differing mechanisms from *Pkd1* and *Pkd2* mutations in ADPKD, *PKHD1* mutations similarly disrupt renal cell signaling and structural integrity, leading to cyst formation primarily in the collecting ducts of the kidneys and often affecting the liver. A notable study identified 77 *PKHD1* variants in 78 ARPKD patients, indicating the gene’s extensive mutational spectrum.^[13]^ Symptoms often present in infancy or early childhood and include enlarged kidneys, hypertension, and varying degrees of renal insufficiency. Liver fibrosis is a common extrarenal manifestation, and affected children may suffer from significant morbidity and mortality related to renal and hepatic complications. ARPKD prognosis is generally poorer, with many affected individuals developing significant renal impairment early in life. Advances in supportive care and potential gene therapies offer hope for improved outcomes, but the disease often leads to ESRD or severe liver disease in childhood or adolescence.

The relationship between genetic mutations and PKD encompasses the disruption of normal protein function, leading to cyst formation and progressive kidney damage. The clinical manifestations and prognosis of the disease depend on the specific genetic mutations involved, with *PKD1* mutations generally resulting in more severe and earlier-onset symptoms compared to *PKD2* mutations in ADPKD, and ARPKD presenting severe symptoms early in life. Advances in gene therapy and other treatments hold promise for altering the course of the disease and improving patient outcomes.

## Classification of gene therapy

Gene therapy has emerged as a transformative approach in modern medical research, leveraging advancements in science and technology to treat or prevent diseases by modifying genes.^[14]^ This innovative field encompasses a variety of strategies based on their unique mechanisms and objectives (Table 1): (1) replacement therapy: This foundational method involves inserting healthy genes into a patient’s cells to replace or augment defective or missing ones, thus restoring normal cellular functions. It’s primarily employed for genetic diseases attributable to single-gene defects; (2) knockout therapy: Utilizing technology to disable or “knock out” specific genes that cause disease, this approach is effective in managing conditions resulting from gene overexpression or mutation, such as certain cancers;^[15]^ (3) repair therapy: Concentrating on the precise correction of gene mutations to reinstate their normal function, this technique represents a direct intervention to rectify genetic abnormalities; (4) enhancement therapy: By introducing specific genes into cells, this method boosts particular cellular functions without replacing or repairing the mutated genes. It is especially useful for treating metabolic disorders by supplementing genes to increase the production of missing or insufficient enzymes or proteins; (5) RNA interference therapy: This strategy employs small RNA molecules to suppress the expression of targeted genes, offering a promising avenue for the treatment of diseases characterized by abnormal gene expression, including cancer and genetic disorders;^[16,17]^ and (6) immunomodulatory therapy: While not a conventional form of gene therapy, genetically engineering patients’ immune cells (*e.g*., through CAR-T cell therapy) to combat cancer cells is also recognized as a gene therapy variant.

**Table 1:** Classification of gene therapy techniques and usage in polycystic kidney disease

Technical classification	Methodologies	Hallmark	Appliance
Viral vector delivery methods	Adenoviral vector	Efficient delivery of genes may cause an immune response	Treatment of hereditary diseases
	Adeno-associated virus vector	Lower immunogenicity, long-term expression potential	Widely used in gene therapy
	Lentiviral vector	Efficient transduction of dividing and non-dividing cells	Treatment of hereditary diseases
	Retroviral vector	Integration into the host genome to provide long-term expression	Certain types of cancer and hereditary diseases
Non-viral delivery methods	Lipid nanoparticles (LNPs)	Encapsulated RNA or DNA for efficient delivery	mRNA vaccines and RNA interference therapy
	Electroporation	Increase in cell membrane permeability by electrical pulses	*In vitro* cellular gene editing
	Other nanoparticles and carriers	Physical or chemical methods to improve delivery efficiency	Disease modeling and treatment research
Gene editing technology	CRISPR/Cas9	High-precision genome editing	Disease modeling and gene therapy
	TALENs	Protein-based targeted gene editing	Targeted modifications of the genome
	ZFNs	Recognizes and cleaves DNA through zinc finger protein structural domains	Knockouts or modifications
RNA technology	RNA interference	Reduction of gene expression by ASO, siRNA, or miRNA	Research and treatment of diseases with abnormal gene expression
	mRNA therapy	Delivery of mRNA induces target protein production	COVID-19 vaccine and treatment of genetic diseases

TALENs: transcription activator-like effector nucleases; ZFNs: zinc finger nucleases; ASO: antisense oligonucleotides.

The advent of CRISPR-Cas9 technology has notably advanced the capabilities of gene editing, providing a mechanism for precise gene modification through the use of guide RNA (gRNA) that directs the Cas9 enzyme to specific DNA sequences for cutting, thereby triggering the cell’s inherent DNA repair mechanisms.^[18]^ This breakthrough has not only revolutionized gene manipulation techniques but also expanded the potential applications of gene editing.

Adeno-associated virus (AAV) vectors have gained prominence as preferred carriers for gene therapy due to their low toxicity, minimal immunogenicity, stable gene expression, and straightforward production process, mitigating the risks associated with gene integration seen in retroviral vectors.^[19]^

In summary, the field of gene therapy offers diverse strategies, each with its specific applications and challenges, including considerations of safety, delivery efficiency, and treatment longevity. The development and application of technologies like viral vectors and CRISPR-Cas9 are pivotal in advancing gene therapy, opening new possibilities for treating a wide range of diseases.

## Advances in hereditary clinical research on gene therapy

The inaugural clinical trials spearheaded by Anderson at the National Institutes of Health (NIH) during the 1990s signified a pivotal moment for gene therapy, transitioning from theoretical constructs to tangible applications in human health. A landmark trial involved infusing genetically modified autologous T cells *via* retroviral vectors into two young girls suffering from severe combined immunodeficiency due to adenosine dehydrogenase deficiency (ADA-SCID).^[20]^ Despite facing substantial challenges, including the refinement of delivery mechanisms, managing immune responses, and enhancing gene transfer efficiency, this trial established a foundational pillar for gene therapy’s potential. Since these early days, significant technological advancements have enabled the approval and commercialization of various gene therapies, offering new avenues of hope for patients with conditions once deemed intractable. Notably, Luxturna’s 2017 approval marked a breakthrough in treating inherited blindness,^[21]^ followed by the 2019 sanctioning of Zolgensma for spinal muscular atrophy, each representing monumental success stories within the gene therapy realm.^[22]^

Recently, a pioneering clinical trial targeting genetic deafness showcased the efficacy and safety of the AAV1-hOTOF vector, delivering the OTOF gene to treat autosomal recessive deafness type 9.^[23]^ Furthermore, gene therapy strategies employing AAV vectors have shown promise in addressing glomerulonephritis by targeting the NPHS2 gene mutation.^[24]^ *In vitro* experiments on human podocytes and subsequent *in vivo* treatments in model mice with an AAV serotype 2/9 vector have demonstrated significant reductions in albuminuria levels and disease progression, underscoring gene therapy’s versatile potential across a spectrum of diseases.^[24]^

In the context of Fabry disease, a clinical trial named FACTs explored the *in vitro* recombinant lentivirus-mediated transfer of alpha-galactosidase A cDNA to treat the condition (NCT02800070). This approach involved reintroducing genetically modified autologous hematopoietic stem/progenitor cells into patients, with the first treatment administered in January 2017.^[25]^ Impressively, no serious adverse events were reported among trial participants, and a two-year study revealed significant enhancements in alpha-galactosidase A activity and reductions in enzyme substrates and metabolites in plasma and urine. These findings suggest that genetically modified hematopoietic stem/progenitor cells hold the capacity for sustained alpha-galactosidase A production, highlighting the innovative strides gene therapy continues to make in treating a broad range of medical conditions.

## Gene therapy for ADPKD

### Antisense oligonucleotides therapy

Abnormally expressed noncoding RNAs (ncRNAs), especially microRNAs (miRNAs), play a key role in the complex pathogenesis of PKD. miRNAs affect cellular functions by regulating the expression of specific protein-coding genes. Recent studies have emphasized the critical role of miRNAs in inhibiting cyst formation, and have proposed the use of miRNAs as a therapeutic new strategy for ADPKD. For example, miR-17-5p can affect the PKD process by specifically targeting the *Pkd1* and *Pkd2* genes, as well as regulating the transcription factor hepatocyte nuclear factor-1 (HNF1), leading to an increase in renal cysts as well as down-regulation of miR-200 family expression.^[26,27]^ In the ADPKD model, decreasing the expression of RNA deconjugated p68 reduced the maturation of specific miRNAs, which in turn inhibits cyst formation.^[28]^ In addition, studies targeting transcription factor-regulated miRNAs overexpressed in PKD have demonstrated the possibility of preventing cyst formation by adjusting miRNA expression.^[29]^

**Figure 1:**
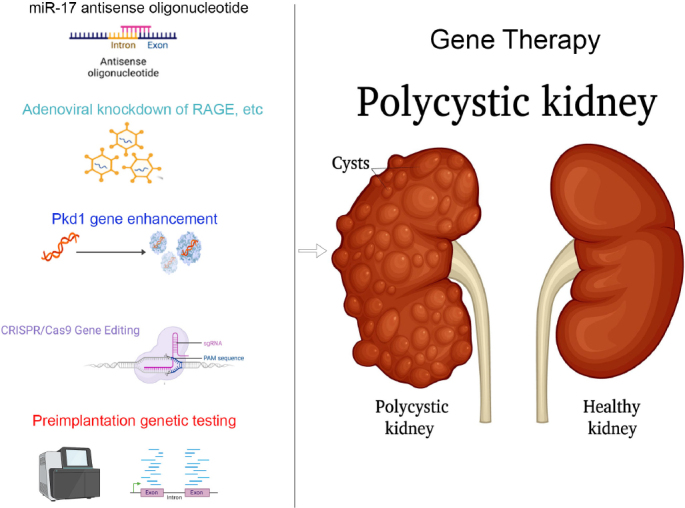
Gene therapies for autosomal dominant polycystic kidney disease.

RGLS4326, an ASO inhibitor targeting miR-17, demonstrated favorable efficacy and safety by subcutaneous administration in an animal model of ADPKD (Figure 1).^[30]^ The drug is preferentially distributed in the kidney. The drug was able to preferentially distribute in cysts in the kidney and collecting ducts, inhibiting miR-17 targets including *Pkd1* and *Pkd2* and effectively controlling cyst growth (Table 2). However, the potential toxicity of RGLS4326 to the central nervous system at high doses prompted researchers to develop RGLS8429 with similar efficacy,^[31]^ but does not affect AMPA receptors, and has been approved by the U.S. Food and Drug Administration (FDA) to initiate a Phase I clinical trial in the second quarter of 2022 (clinicaltrials.gov#NCT05429073), which is currently ongoing.

**Table 2:** Applications, advantages, and disadvantages of each gene therapy technique in polycystic kidney disease

Disease	Treatments	Methods	Target	Status	Advantages	Disadvantages
ADPKD	ASO (RGLS4326)	Subcutaneous injection by lipid nanoparticles	microRNA-17	Clinical trial	Specific targeting of microRNA-17	Potential injection site reactions
	Genetic blockade	PGT in combination with *in vitro* fertilization	*PKD1/2*	Clinical trial	Prevention of disease transmission	Ethical and technical challenges
	shRNA	Adenovirus vector	*RAGE*	Animal study	Effective gene silencing	Potential for immune response
	Transgenic mice	Inducible gene enhancement in embryo	*PKD1*	Animal study	Long-term study of gene function	Ethical considerations, complexity
	Transgenic mice	Inducible gene enhancement in embryo	PC1-CTT	Animal study	Long-term study of gene function	Ethical considerations, complexity
	Gene editing	Elimination of miR-17 binding element in 3’-UTR by CRISPR/Cas9	*PKD1*^Δ*17*^/*PKD2*^Δ*17*^	Animal study	Enhances mRNA stability and protein levels, retards cyst growth	Off-target effects, potential for unintended gene regulation changes
ARPKD	ASO (AVI-4126, Eteplirsen)	Intravenous infusion by lipid nanoparticles	*c-myc*	Clinical trial	Potential for systemic delivery	Potential for immune response, cost
	Gene editing	CRISPR/Cas9	*P2rx7*	Cell experiment	High-precision gene editing	Off-target effects, technical challenges

ASO: antisense oligonucleotides; RAGE: receptor of advanced glycation end product; PGT: preimplantation genetic testing.

### Adenoviral knockdown of RAGE inhibits cystogenesis in ADPKD

The receptor of advanced glycation end product (RAGE) is transmembrane, and existing studies have shown that RAGE mediates the activation of intracellular signaling pathways, participates in inflammatory responses, and promotes cell proliferation and survival-related signaling. Researchers transduced anti-RAGE shRNA into ADPKD mice *via* adenoviral vectors and demonstrated that kidney size, cystogenesis, and renal function were improved in ADPKD mice with downregulation of the RAGE gene.^[32]^ These results suggest that the RAGE-related signaling pathway is closely related to the pathogenesis of PKD, and the RAGE gene may be a new potential therapeutic target for PKD.

### Pkd1 gene enhancement therapy in vivo

Previous studies have suggested that a decrease in *Pkd1* expression levels below a critical threshold can lead to the formation of renal cysts and other clinical signs of ADPKD.^[33]^ Transfer of *Pkd1/2* through the germ line to *Pkd1* null mice may be effective, for *Pkd1/2* conditionally inactivated at weeks 4-6. Kurbegovic *et al*. found that constructing full-length genomic *Pkd1* into a *Pkd1*-inactivated mouse model rescued *Pkd1-deficient* mice.^[34]^ According to what Kurbegovic *et al*. found in their study, the introduced high copy number renal targeting genes*^SB^ Pkd1* and *Pkd1^Minigene^* showed expression levels similar to those of the endogenous *Pkd1* gene, produced functional PC1 proteins *in vivo* that delayed or even eliminated renal cyst formation, and extended lifespan by up to 4-fold to completely rescue PKD mice. This evidence provides theoretical support for the use of first-generation *Pkd1* gene transfer for gene enhancement therapy. The gene enhancement approach may be a more promising and appropriate therapeutic strategy for PKD patients with reduced PC1 doses. Recently, Laura *et al*.^[35]^ demonstrated that transgenic expression of the C-terminal tail of PC1 in ADPKD mouse models suppressed the cystic phenotype and preserved renal function. This finding indicates that reexpressing polycystin or a truncated version of the protein in ADPKD could be a promising therapeutic approach worth further investigation.

### Curing ADPKD by spontaneous gene repair behavior in Pkd1 mutant iPSCs

The technology of induced pluripotent stem cells (iPSCs) has shown potentially revolutionary promise in therapeutic research for ADPKD.^[36]^ iPSCs allow scientists to study the effects of specific genetic mutations and their possible repair by epigenetically reprogramming an individual’s somatic cells into stem cells. It was found that through spontaneous mitotic recombination, iPSCs with mutations in the *Pkd1* gene can achieve genetic repair, transforming from *Pkd1*(+/-) to *Pkd1*(+/R+). This genetic repair resulted in iPSCs that were genotypically not significantly different from healthy cells. Further studies showed that adult chimeric mice derived from iPSCs containing the repaired *Pkd1* gene (+/R+) had a significantly lower frequency of renal cyst formation than mice derived from iPSCs containing the original *Pkd1*(+/-) mutation and were not significantly different from normal mice.^[36]^ This provides a new therapeutic avenue for genetic diseases such as ADPKD, where genetic defects are corrected by mitotic recombination-mediated gene repair. This study not only reveals the potential of iPSCs in understanding and treating ADPKD but also opens up new avenues for future clinical applications using human induced pluripotent stem cells. By precisely correcting genetic mutations, we may eventually be able to provide more effective and personalized treatment options for ADPKD patients.

### CRISPR/Cas9 therapy in ADPKD

CRISPR/Cas9 technology has shown great promise in the therapeutic field of genetic diseases, including ADPKD. One promising approach involves stabilizing *PKD1*/2 mRNA translation to alleviate ADPKD symptoms.^[37]^ Research has demonstrated that mRNAs produced by the noninactivated *PKD1* allele are repressed *via* their 3′-UTR miR-17 binding element. Eliminating this motif (*Pkd1*^Δ17^) improves mRNA stability, raises Polycystin-1 levels, and alleviates cyst growth in cellular, ex vivo, and mouse PKD models.^[37]^ Similarly, *Pkd2* is inhibited *via* its 3′-UTR miR-17 motif, and *Pkd2*^Δ17^-induced Polycystin-2 derepression retards cyst growth in Pkd1-mutant models.^[37]^ These findings suggest that evading 3′-UTR cis-interference and enhancing *PKD1*/2 mRNA translation is a potentially mutation-agnostic approach to arresting ADPKD.

Additionally, CRISPR/Cas9 has been widely used in gene therapy research for constructing PKD models and disease models. Through CRISPR-Cas9 and somatic cell cloning, Masahito *et al*. successfully created a pig model with a specific mutation *Pkd1i*^*nsG/*+^.^[38]^ Pathological analyses of the primary and offspring of the cloned animals showed that *Pkd1*^*insG/*+^ pigs have many similar characteristic manifestations to human ADPKD patients, providing an ideal model for in-depth studies of ADPKD pathogenesis and therapeutic approaches. In addition, Romano *et al*. successfully generated a series of iPSCs carrying heterozygous mutations or compound heterozygous mutations in the *Pkd1* gene using CRISPR-Cas9 technology (*Pkd1*^+/-^ and *Pkd1*^-/-^).^[39]^ These iPSCs maintained the morphology, normal karyotype, pluripotency, and differentiation capacity of stem cells during differentiation into trichoblasts, further demonstrating the effectiveness of CRISPR technology in mimicking and investigating the pathogenesis of PKD. These applications of CRISPR/ Cas9 technology not only demonstrated its great potential in the therapeutic field of genetic diseases but also pointed to the challenges faced by the current technology, including how to reduce off-target effects and meet the need for customization of treatments for specific ADPKD family line populations. As the technology continues to advance and be optimized, CRISPR/Cas9 is expected to provide a more precise and safer strategy for the treatment of PKD and other genetic diseases.

### PGT blocks ADPKD inheritance

Preimplantation genetic testing (PGT) is a prenatal genetic diagnosis that allows early identification of abnormal embryos. PGT in combination with assisted reproduction technology (ART) procedures can lead to genetically normal embryos. The first successful application of PGT in combination with *in vitro* fertilization (IVF) was reported in 1990. Since then, the technique has been progressively applied to a wide range of genetic disorders. Our previous research studies have shown that patients with fertility intentions represent approximately 45.0% of the overall age-appropriate ADPKD population, and the majority of these patients (79.6%) are willing to use the PGT technique.^[40]^ In recent years, with the development of a new genome-wide amplification method, MALBAC, the sensitivity of singlecell detection of PKD mutations has been dramatically improved, effectively ensuring the accuracy of PGT. We first used MALBAC-PGT combined ART on an ADPKD couple in 2015, and a healthy baby was successfully born in 2016.^[41]^ To further investigate the efficacy, safety, and long-term effects on offspring using PGT combined with ART in ADPKD patients, we organized and implemented this multicenter clinical cohort study with long-term followup.^[42]^ The trial (ESPERANCE) is available at clinicaltrials. gov (NCT 02948179) and completed in 2023. A total of 711 ADPKD patients were enrolled for genetic counseling in 54 departments of 27 tertiary hospitals across China (Nephrology Department combined with the Center for Reproductive Medicine), of which 459 patients agreed to undergo sequencing of the Pkd1/2 gene. Although the choice of PGT depends on the individual decision of the ADPKD patient, we suggest that PGT should be widely promoted as a prioritized reproductive option in genetic counseling for ADPKD patients with reproductive intent. This technique will result in fewer and fewer ADPKD patients worldwide, significantly reduce the medical burden on families and society, and result in a higher-quality birth population.

## Gene therapy for ARPKD

### Antisense oligonucleotide therapy for ARPKD

Antisense oligonucleotide technology has also demonstrated great potential in therapeutic studies of ARPKD. By mediating the correction of mRNA shearing, specifically targeting about 7.7% of splicing variants in the *PKHD1* gene, ASO provides a new avenue for the treatment of ARPKD caused by splicing defects. This strategy can correct aberrant gene splicing events and increase the expression of the normal *PKHD1* gene, opening up new possibilities for the treatment of ARPKD caused by splicing defects.^[43]^ Enhanced renal *c-myc* mRNA expression has been observed not only in rodent animal models of PKD but also in human ADPKD patients. It was found that treatment of ARPKD mouse models with antisense oligonucleotides targeting *c-myc was* effective in improving disease symptoms.^[44]^ AVI BioPharma is developing AVI-4126, an antisense oligonucleotide aimed at c-myc mRNA, for the treatment of restenosis, cancer, and polycystic kidney disease.^[45]^ This drug is currently in phase II clinical trials.^[45]^ These findings emphasize the critical role of *c-myc* in PKD cyst formation and present antisense oligonucleotide targeting of *c-myc* as a potential new therapeutic strategy.

### CRISPR/Cas9 knockdown of P2rx7 gene delays cyst growth in vivo

Early experiments revealed the key role of P2X receptors, especially P2X7, in PKD cyst formation. In ARPKD and ADPKD model mice, the protein expression level of the P2X7 receptor was significantly elevated in cystic epithelial cells.^[46,47]^ Pannexin-1 (PANX-1), an ion channel capable of releasing ATP, is also significantly more expressed in cystic epithelial cells than in normal tubular epithelial cells. Activation of P2X7 signaling promotes PANX-1-mediated release of ATP into the lumen, while decreasing sodium reabsorption in the cyst wall, ultimately leading to hyperproliferation of cystic epithelial cells. Using CRISPR/Cas9 technology, Arkhipov *et al*. performed global knockdown of the *P2rx7* gene in an ARPKD rat model and found that cyst growth was significantly slowed down in ARPKD rats after knockdown of the *P2rx7* gene compared to controls carrying the *P2rx7* gene. This finding suggests that a new pathway for PKD treatment may be opened by targeting *P2rx7*.^[48]^

## Challenges and prospects

Although gene editing and RNA interference technologies have shown great potential in laboratory research, there are still significant challenges in translating these technologies into safe and effective clinical treatments. The technical challenges mainly include improving editing efficiency, ensuring editing precision to minimize off-target effects, and developing efficient delivery systems to ensure that therapeutic molecules can reach and enter target cells. For example, the development of miRNA therapeutics faces technical limitations in improving drug delivery mechanisms and molecular stability. Effective delivery of therapeutic genes to the kidney is a major challenge in current gene therapy research, and existing gene therapy vectors such as AAVs and lipid nanoparticles (LNPs) have limited efficacy when targeting the kidney. The complex structure of the kidney and the glomerular filtration barrier pose challenges for targeting specific renal cell types. In PKD, this means targeting the renal tubular epithelial cells where cysts form. Ensuring specificity, achieving efficient delivery, and minimizing off-target effects are crucial for successful targeting.

The choice of vector is critical in gene therapy, as it influences the efficiency, safety, and duration of gene expression. Commonly used vectors in PKD gene therapy research include AAV vectors, which have low immunogenicity and the ability to infect both dividing and non-dividing cells but face challenges like limited cargo capacity and pre-existing immunity. Perhaps using two or more AAV vectors could successfully deliver the entire Pkd1 gene. Lentiviral vectors, known for their high efficiency of gene transfer, pose risks of insertional mutagenesis. The CRISPR-Cas9 system offers high precision in gene editing but requires efficient delivery mechanisms and strategies to minimize immune responses.

To this end, scientists are exploring new ways to deliver therapeutic genes directly to the kidney, including retrograde ureteral injections, subcapsular injections, renal artery injections, and retrograde renal vein injections, to achieve precision therapies in an in situ setting. The route of administration is crucial for ensuring that therapeutic agents reach the kidneys in sufficient concentrations. Intravenous (IV) injection offers a non-invasive option but may struggle to achieve high concentrations in the kidneys. Direct delivery methods like retrograde ureteral injection, subcapsular injection, and renal artery injection provide higher local concentrations but are more invasive and technically challenging.

Moreover, a potential immune response is an issue to overcome for CRISPR/Cas9 technology.CRISPR/Cas9 gene editing may trigger p53-mediated DNA damage responses in some cases.^[49]^ Safety issues of gene therapy, especially potential long-term side effects, are key factors that must be considered when moving to clinical applications.

The application of gene-editing technologies raises ethical issues, particularly concerning the boundaries of genetic modification, how to ensure that these technologies are not used for non-medical purposes given the current tentative absence of a regulatory system, and so on. Social acceptance of these technologies and the establishment of relevant legal and ethical guidelines will be an important part of the development of gene therapy. For the time being, researchers need to be extra vigilant on how to safeguard the rights of subjects from being violated, while avoiding the subsequent incalculable problems associated with gene therapy. In addition, the high cost of gene therapy makes these potential treatments potentially difficult for a wide range of patients to accept. How to ensure that these treatments are available to patients globally, especially in resource-limited regions, is therefore another major challenge.

As gene editing and RNA interference technologies continue to advance, the future holds the promise of solving the technical challenges currently faced. For example, the precision and efficiency of gene editing can be improved by improving the design of CRISPR systems and developing new delivery methods. Bioinformaticians have invented a series of computational tools to optimize CRISPR systems, helping researchers design more efficient gRNAs to reduce off-target effects.^[50]^ Next-generation gene editing tools, such as variants of CRISPR and entirely new editing systems, may provide safer and more effective therapeutic options. The development of gene therapy will accelerate the realization of personalized medicine. By analyzing an individual’s genetic information, therapeutic strategies can be designed to target specific genetic variants, thus providing more precise treatment. This will not only improve the effectiveness of treatment but also help reduce unwanted side effects. Addressing the challenges facing gene therapy requires multidisciplinary collaboration. Experts in the fields of biology, medicine, engineering, ethics, and law need to work together to advance the technology and ensure safe and ethical treatments, as well as to pave the way for the commercialization and diffusion of gene therapy. As global awareness of the importance of treating genetic diseases increases, the field of gene therapy will receive more support and investment in research and applications. The establishment of international collaborative programs and global research networks will help accelerate the development and application of gene therapy technology to benefit patients worldwide.

In conclusion, although gene therapy for PKD faces many challenges on the road to achieving clinical application, it offers unprecedented opportunities for the treatment of genetic diseases as science and technology advance and global collaboration deepens. In the future, gene therapy has the potential to revolutionize the way we treat diseases and bring new hope to patients with PKD as well as other genetic diseases.
